# Quantitative methods for assessing local and bodywide contributions to *Wolbachia* titer in maternal germline cells of *Drosophila*

**DOI:** 10.1186/s12866-019-1579-3

**Published:** 2019-09-03

**Authors:** Steen Christensen, Moises Camacho, Zinat Sharmin, A. J. M. Zehadee Momtaz, Laura Perez, Giselle Navarro, Jairo Triana, Hani Samarah, Michael Turelli, Laura R. Serbus

**Affiliations:** 1 Department of Biological Sciences, Florida International University, Miami, FL 33199 USA; 2 Biomolecular Sciences Institute, Florida International University, Miami, FL 33199 USA; 3 Department of Evolution and Ecology, University of California, Davis, Davis, CA 95616 USA

**Keywords:** Colonization, Titer, Quantification, Imaging, qPCR, Endosymbiont, *Wolbachia*, *Drosophila*, Germline, Oogenesis

## Abstract

**Background:**

Little is known about how bacterial endosymbionts colonize host tissues. Because many insect endosymbionts are maternally transmitted, egg colonization is critical for endosymbiont success. *Wolbachia* bacteria, carried by approximately half of all insect species, provide an excellent model for characterizing endosymbiont infection dynamics. To date, technical limitations have precluded stepwise analysis of germline colonization by *Wolbachia.* It is not clear to what extent titer-altering effects are primarily mediated by growth rates of *Wolbachia* within cell lineages or migration of *Wolbachia* between cells.

**Results:**

The objective of this work is to inform mechanisms of germline colonization through use of optimized methodology. The approaches are framed in terms of nutritional impacts on *Wolbachia*. Yeast-rich diets in particular have been shown to suppress *Wolbachia* titer in the *Drosophila melanogaster* germline. To determine the extent of *Wolbachia* sensitivity to diet, we optimized 3-dimensional, multi-stage quantification of *Wolbachia* titer in maternal germline cells. Technical and statistical validation confirmed the identity of *Wolbachia* in vivo*,* the reproducibility of *Wolbachia* quantification and the statistical power to detect these effects. The data from adult feeding experiments demonstrated that germline *Wolbachia* titer is distinctly sensitive to yeast-rich host diets in late oogenesis. To investigate the physiological basis for these nutritional impacts, we optimized methodology for absolute *Wolbachia* quantification by real-time qPCR. We found that yeast-rich diets exerted no significant effect on bodywide *Wolbachia* titer, although ovarian titers were significantly reduced. This suggests that host diets affects *Wolbachia* distribution between the soma and late stage germline cells. Notably, relative qPCR methods distorted apparent *wsp* abundance, due to altered host DNA copy number in yeast-rich conditions. This highlights the importance of absolute quantification data for testing mechanistic hypotheses.

**Conclusions:**

We demonstrate that absolute quantification of *Wolbachia,* using well-controlled cytological and qPCR-based methods, creates new opportunities to determine how bacterial abundance within the germline relates to bacterial distribution within the body. This methodology can be applied to further test germline infection dynamics in response to chemical treatments, genetic conditions, new host/endosymbiont combinations, or potentially adapted to analyze other cell and tissue types.

**Electronic supplementary material:**

The online version of this article (10.1186/s12866-019-1579-3) contains supplementary material, which is available to authorized users.

## Background

The mechanisms by which bacteria colonize eukaryotic cells are of central interest to diverse biological disciplines, as well as biomedical and health practice [1–3]. Horizontal invasion mechanisms, such as non-selective uptake of nutrients and antigens into large, endocytic vacuoles, continue to be investigated in depth, particularly with respect to bacterial pathogens [4, 5]. Vertical transmission mechanisms, as in the inheritance of bacteria by daughter cells during mitosis, also play a key role in transmission of bacterial endosymbionts [6–8]. Following bacterial entry into eukaryotic cells, subsequent rounds of bacterial replication continue the colonization process, which concludes in cessation of bacterial replication or egress of bacteria through exocytosis and/or host cell lysis [9–11]. We do not know the relative roles of bacterial loading and replication within host cells, nor bacterial movement between host cells in determining *Wolbachia* titer.

The extent to which colonization mechanisms are shared between pathogenic and non-pathogenic bacteria is also unclear. Bacterial endosymbionts are carried by diverse host taxa, with dozens having been identified in insects alone [12]. Endosymbiotic *Wolbachia* bacteria are carried by approximately 50% of all insect species, as well as some mites, crustaceans and filarial nematodes [13–16]. In the majority of host organisms, *Wolbachia* are regarded as facultative, often, but not always [17], producing reproductive manipulation [18, 19]. *Wolbachia* are maternally transmitted, with infection of germline cells ultimately loading the bacteria into eggs. Studies in the *Drosophila melanogaster* germline have the advantages of a well-developed model system and a natural *Wolbachia* infection. As such, this system is expected to provide a model for physiological mechanisms of *Wolbachia* colonization [20–24].

**Fig. 1:**
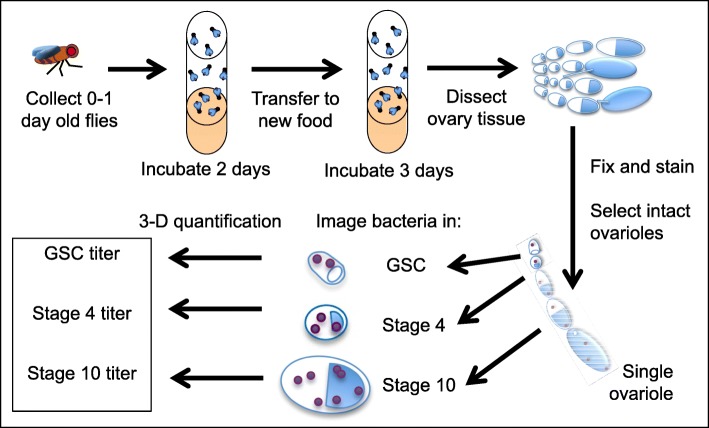
Approach used for *Wolbachia* titer analysis in *D. melanogaster* oogenesis. The workflow is presented for fly preparation, tissue processing, ovariole selection, and image analysis. Morphology and position of the oocyte were among the criteria used for staging individual egg chambers. At the distal tip of the ovariole: blue represents the germline stem cell. At stage 4 and stage 10: half blue, half white: ovals represent individual egg chambers. The blue section represents the oocyte. Shown as purple dots: germline *Wolbachia*

Organization of the *D. melanogaster* maternal germline makes it particularly amenable to studies of endosymbiont colonization. Developing eggs are formed within 16–23 structured ovary subunits termed “ovarioles” [25] (Fig. 1). Within each ovariole, germline stem cells (GSCs) are juxtaposed against terminal filament cells at the distal tip of the structure [26–28]. Daughter cells produced from the GSC undergo 4 rounds of cell division with incomplete cytokinesis to form an interconnected cyst of germline cells. The resulting 16-cell cyst, coated with a layer of somatic follicle cells, is referred to as an egg chamber. These egg chambers go through 14 developmental stages over three and a half days to produce a completed egg [26]. These developmental stages are presented in order of age, with the youngest positioned at the ovariole anterior, and oldest toward the ovariole posterior, due to the intrinsic tubular structure of the ovariole (Fig. 1). Thus, examination of *Wolbachia* in *D. melanogaster* ovarioles provides staged windows into the timeline of colonization by *Wolbachia.*

Studies of ovary colonization by *Wolbachia* have employed a range of cytological approaches across arthropod and nematode host systems. Researchers have used DNA dyes [24, 29–35], anti-*Wolbachia* surface protein (WSP) antibodies [36–39], anti-Hsp 60 antibodies [20, 22, 31, 40, 41], and fluorescence in situ hybridization [21, 42–47]. In *D. melanogaster* oogenesis, these staining methods have revealed *Wolbachia* carried in maternal GSCs and their daughter cells, demonstrating *Wolbachia* transmission during mitosis [13, 31, 48]. There is also evidence that *Wolbachia* can horizontally invade newly forming cysts [43] and early-mid stage egg chambers [29]. *Wolbachia* also divide via binary fission in the germline [43, 49]. The combined inputs from mitotic inheritance, cell-cell migration and replication within host cells are estimated to result in *Wolbachia* loads on the order of 3000–18,000 bacteria per egg [50, 51].

What remains unclear is the extent to which initial load, horizontal invasion, and bacterial replication contribute to the ultimate number of bacteria carried by the egg. Because existing stains have not provided uniformly crisp resolution of *Wolbachia* bacteria across oogenesis, this has precluded systematic, quantitative analyses. This technical shortcoming curtails mechanistic understanding of germline *Wolbachia* loads. Quantitative analyses of *Wolbachia* titer have been restricted to one or a subset of developmental stages, for the purpose of addressing how candidate host factors affect *Wolbachia* loads. Studies of developmental [49], cytoskeletal [21, 24, 31, 52] and nutritional impacts [53, 54] on germline *Wolbachia* titer have provided initial insights. However, without understanding the timeline of colonization, we cannot interpret observed changes in *Wolbachia* density.

The story of host dietary impact on germline *Wolbachia* serves as an example of how limitations to date are resolved using optimized methodology. We previously found that stage 10 egg chambers exhibited striking depletions of *Wolbachia* from adult flies that ate yeast-enriched food [53]. It is known that yeast drives neural insulin-producing cells (IPCs) to release insulin-like peptides into the hemolymph [55]. A series of experiments, including ablation of neural IPCs ultimately demonstrated that yeast-driven insulin release suppresses germline *Wolbachia* abundance, referred to as “titer” [53]. The basis for this titer reduction was unclear, however, with no information available from other stages of development, nor from germline vs. bodywide comparisons. The methods presented here can resolve these questions, as described below. Optimized cytological approaches provide insight into *Wolbachia* titer at timepoints spanning 95% of maternal germline development. In this study, the data show that yeast diets do not induce a cumulative bacterial loading deficiency in oogenesis, rather, germline *Wolbachia* titers are diet-sensitive during late oogenesis. Furthermore, optimized quantification of *Wolbachia* by absolute qPCR enables tracking of *Wolbachia* titers across whole fly and ovarian samples. The data demonstrated that ovarian *Wolbachia* titers are diet-sensitive, whereas whole-body *Wolbachia* titers are not. Technical and statistical validation supports the mechanistic insights yielded by these methods: the implication that late oogenesis is subject to diet-sensitive redistribution of *Wolbachia* between germline and soma.

## Results

### DNA staining of cytosolic nucleoids across oogenesis represents *Wolbachia*

**Fig. 2:**
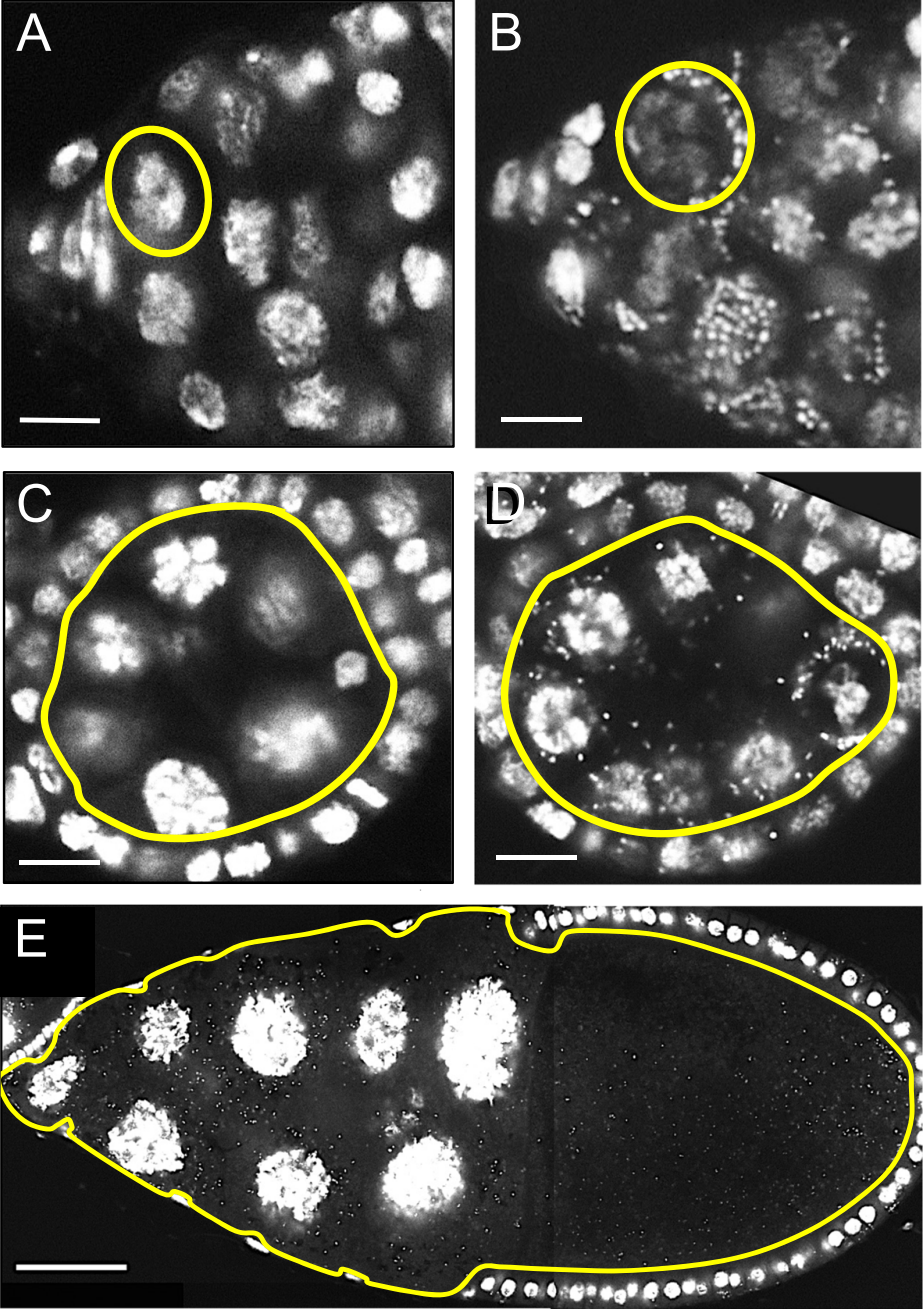
*Wolbachia* labeling in oogenesis by propidium iodide. Host DNA is visible as large circles, and *Wolbachia* as small puncta. Yellow outlines: germline cells. **a** *Wolbachia*(−) GSC. **b** *Wolbachia*(+) GSC. **c** *Wolbachia*(−) stage 4 germline cyst. **d** *Wolbachia*(+) stage 4 germline cyst. **e** *Wolbachia*(+) stage 10 germline cyst. Nurse cells left, oocyte right. Scale bars: **a-d** 5 μm. **e** 50 μm

To systematically assess *Wolbachia* titer in maternal germline cells, we analyzed *Wolbachia* load at specific timepoints of oogenesis. Ovarian tissues were dissected from *D. melanogaster* females that carried the *w*Mel strain of *Wolbachia* (Fig. 1) [56]. Tissues were fixed according to a modified TUNEL staining protocol [57] and labeled with propidium iodide. Ovarioles that each carried discernable germline stem cells (GSCs), a stage 4 egg chamber, and a stage 10 egg chamber [26, 27] were imaged by confocal microscopy (Fig. 1) (Fig. 2). Fly stocks confirmed as *Wolbachia*(*+*) by PCR also showed defined DNA staining foci in the cytoplasm of germline cells at all selected stages (Fig. 2b, d, e). By contrast, fly stocks indicated as *Wolbachia*(*−*) by PCR did not exhibit any punctate cytoplasmic staining (Fig. 2a, c). The correlation of cytoplasmic DNA staining puncta with *Wolbachia* detected by standard PCR suggests that these puncta represent *Wolbachia* nucleoids.

**Fig. 3:**
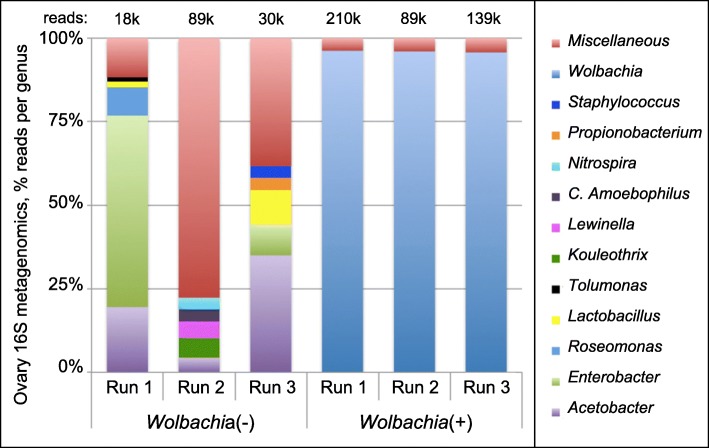
16S microbiome profiles associated with *Wolbachia*(−) and *Wolbachia*(+) ovaries. Shown: top 5 most abundant genra that represented > 1% of reads. Further detail presented in Additional file 2: Table S3 and Data Files S1–6

Despite PCR confirmation of *Wolbachia* in germline cells, the extent to which DNA staining detects other microbes is unknown. To resolve this, deep sequencing of bacterial 16S ribosomal RNA genes was performed on ovarian tissues dissected from *Wolbachia*(*−*) and *Wolbachia*(*+*) flies. With additional amplification required for 2 out of 3 *Wolbachia*(−) samples, the 16S rRNA amplicon analyses ultimately returned 18,000–89,000 reads, presumably representing low-abundance bacterial contaminants. Predominant taxa included *Acetobacter* and *Enterobacter,* analogous to gut microbiomes reported previously (Fig. 3) (Additional file 2: S1–S6) [58, 59]. By contrast, standard amplification of *Wolbachia*(*+*) samples yielded between 89,000–209,000 bacterial 16S rRNA amplicon reads, with 94–97% attributed to *Wolbachia* (Fig. 3) (Additional file 2: Table S1) (Additional file 3: S1–6)*.* The large difference in the composition of reads between *Wolbachia*(−) and *Wolbachia*(+) fly strains confirms *Wolbachia* as the primary identity of DNA staining puncta observed in *D. melanogaster* germline cells.

### Multi-stage titer analyses inform baseline progression of colonization in oogenesis

**Fig. 4:**
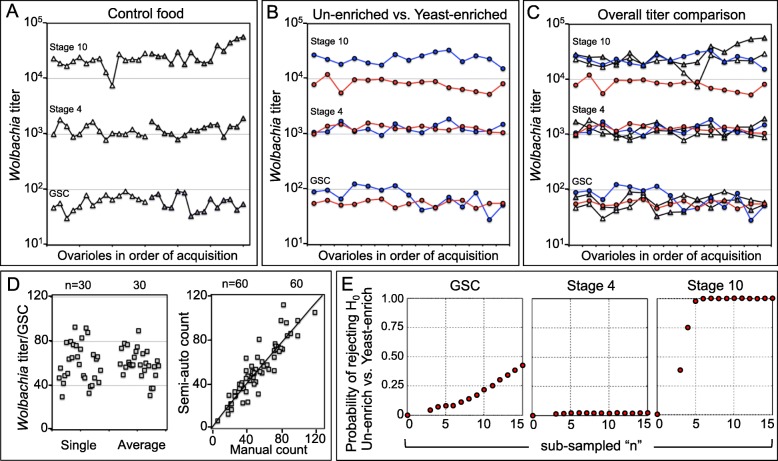
Analysis of *Wolbachia* titer across oogenesis. GSC: Germline stem cell. **a** Titer data, displayed by stage and order of ovariole acquisition. White: First 15 ovarioles imaged. Grey: Last 15 ovarioles imaged. **b** Germline titer data in response to nutritional conditions. Blue: Un-enriched control. Red: Yeast-enriched treatment. **c** Overlay of all 3-dimensional titer data, in groups of 15 ovarioles. **d** Left: Comparison of control titer data for single GSCs versus median GSC titers scored in GSC clusters. Right: Comparison of data acquired by semi-automated versus manual assessment methods. Black line indicates regression analysis. **e** Comparing randomly sub-sampled data from un-enriched control and yeast-enriched treatment conditions. 10,000 iterations determined the frequency of significance, with alpha set at 0.01 (n = 3–15 ovarioles). Tests used: Independent T-tests: GSCs and stage 10. Mann-Whitney U: stage 4

To analyze the process of germline colonization by *Wolbachia,* we performed 3-dimensional imaging of single GSCs, stage 4 germline cysts, and stage 10 germline cysts on *Wolbachia*(+) flies reared on control food (Additional file 1: Figure S1). Manual quantification of *Wolbachia* yielded median values of 61.5 *Wolbachia* puncta per GSC and approximately 1140 in stage 4 cysts (n = 30) (Additional file 2: Table S2). Manual quantification was not possible for late stage germline cells due to the high abundance of *Wolbachia* [49]. Semi-automated quantification of stage 10 germline cysts yielded a median titer of approximately 22,500 *Wolbachia* (n = 30) (Fig. 4a) (Additional file 1: Figure S1) (Additional file 2: Table S2). Consistent with prior work, these data demonstrated significant *Wolbachia* titer increases across oogenesis (Kruskal-Wallis ANOVA *p* < 0.001; n = 30) (Additional file 2: Table S3) [31, 49, 60].

To evaluate the efficacy of cytological *Wolbachia* quantification throughout development, we compared estimates based on alternative methods. As ovarioles typically carry 2–3 GSCs, we compared titer values from single GSCs to titer estimates derived from GSCs clusters. Analysis of GSC clusters showed a median titer of 58.9 *Wolbachia* nucleoids per GSC, not significantly different from 61.5 *Wolbachia* per single GSC (Welch’s T-test *p* = 0.878) (n = 30) (Fig. 4d) (Additional file 2: Table S2-Table S4) [60, 61]. We also compared manual and semi-automated *Wolbachia* scoring methods. *Wolbachia* recounts were performed on selected regions of consistent size, derived from stage 10 oocyte images. No significant difference was detected between *Wolbachia* titer values from manual and semi-automated scoring methods (T-test *p* = 0.896) (n = 60) (Additional file 2: Table S3 and Table S5). Regression analysis yields R^2^ = 0.854 (Fig. 4d). This supports the technical consistency of methods for assessing germline *Wolbachia* titer.

The reproducibility of *Wolbachia* titer profiles within ovarioles was also examined under standard food conditions. To do this, *Wolbachia* titers at each developmental stage were plotted by ovariole (n = 30) (Fig. 4a). Some variation in *Wolbachia* titer was associated with each developmental stage, and particularly evident at stage 10. However, *Wolbachia* titer variation did not present as trends within each ovariole (Fig. 4a). Regression analyses failed to identify a correlation between *Wolbachia* titers of GSCs and stage 4 (R^2^ = 0.108), between stage 4 and stage 10 (R^2^ = 0.159), nor between GSCs and stage 10 (R^2^ = 0.084) (n = 30). This lack of titer correlation between developmental stages suggests that, despite the shared environment of an ovariole, germline titer at earlier stages does not predict titer at later stages. Rather, this analysis shows that each egg chamber represents a distinct instance of colonization.

### Staged analyses show *Wolbachia* titer sensitivity to dietary yeast in late oogenesis

Germline *Wolbachia* titer is known to be responsive to host diet. Specifically, exposing 2-day old adults to yeast-rich diets for 3 days reduces titer in single-focal-plane analyses of stage 10 germline cells [53, 54]. To determine whether this effect is generalized to oogenesis, *Wolbachia* titer analyses were performed on adults exposed to control versus yeast-enriched conditions, referred to as “un-enriched” and “yeast-enriched”, respectively, from this point forward (Additional file 3: S7). Median GSC titer from the un-enriched control was 79.0, as compared to 55.0 in the yeast-enriched treatment (Welch’s T-test *p* = 0.017, n = 15) (Fig. 4b) (Additional file 2: Table S3 and Table S6). Sub-sampling of the data showed an approximate 40% chance of significance with the α-value set at 0.01 when sampled at n = 15 (Fig. 4e). However, examining the data in order of acquisition reduces certainty in GSC titer responses to diet. *Wolbachia* titer in the first acquired un-enriched control images was significantly different from yeast-enriched images acquired in parallel (T-test *p* < 0.001, n = 8) (Fig. 4b) (Additional file 2: Table S3). In contrast, *Wolbachia* titer in the latter acquired un-enriched images was not significantly different from the yeast-enriched treatment run in parallel (T-test *p* = 0.846, n = 8), nor from sub-samples of the initially acquired control GSC values (Welch’s T-test, *p*-value range: 0.216–0.588, n = 15) (Fig. 4c) (Additional file 2: Table S3). Thus, the response of GSC *Wolbachia* titer to yeast-enriched, nutrient-altered diets remains unclear.

*Wolbachia* titer, as quantified by these methods, showed strong sensitivity to host diet in late oogenesis, but not by stage 4. A median of 1180 *Wolbachia* was detected in the un-enriched control, as compared to 1260 in the yeast-enriched treatment (Mann-Whitney *p* = 0.567, n = 15) (Fig. 4b) (Additional file 2: Table S3 and Table S6) [61, 62]. By contrast, yeast-treated stage 10 cysts carried *Wolbachia* loads only 36% those of un-enriched controls, as indicated by a median *Wolbachia* titer of 8240 in the yeast-enriched treatment versus 22,900 in un-enriched control (Welch’s T-test *p* < 0.001, n = 15) (Fig. 4b) (Additional file 2: Table S3 and Table S6). Sub-sampling of the data further supports these statistical interpretations. The probability of significance at an α-value of 0.01 remained approximately 2% for stage 4 regardless of sample size (range: n = 3 to n = 15), whereas comparable power was achieved at stage 10 by analyzing as few as 6 egg chambers (Fig. 4e). Direct examination of the data confirmed the stage-specific titer responses to host diet. *Wolbachia* titer measurements from un-enriched and yeast-enriched conditions overlapped extensively at stage 4, but very little at stage 10 (Fig. 4c). Thus, results yielded by this methodology demonstrate that *Wolbachia* titer suppression by dietary yeast is restricted to later developmental stages and not generalized to whole ovarioles.

### Refined qPCR analyses show body-wide *Wolbachia* titers are insensitive to dietary yeast

**Fig. 5:**
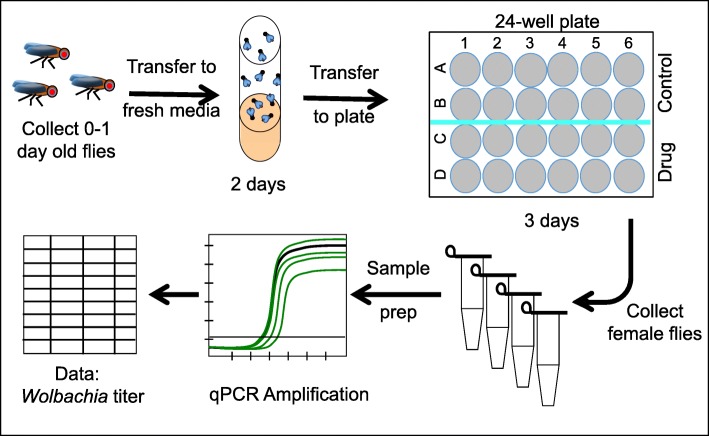
Approach used for real-time quantitative PCR analysis of *Wolbachia* titer in whole *D. melanogaster* flies. The workflow used for fly preparation, drug treatment, sample preparation and qPCR analysis is shown

The cytological data indicate that *Wolbachia* titers are differentially sensitive to host diet across oogenesis. This disparity opens the broader question of whether body-wide *Wolbachia* loads respond to host nutrition. To investigate this, we used quantitative PCR to analyze body-wide gene copy number of a *Wolbachia*-specific marker, the *wolbachia surface protein (wsp)* gene (Fig. 5). The absolute quantification method was used, in which *wsp* copy number amplified from experimental samples is compared against known concentrations of a plasmid standard [56, 63–67].

**Fig. 6:**
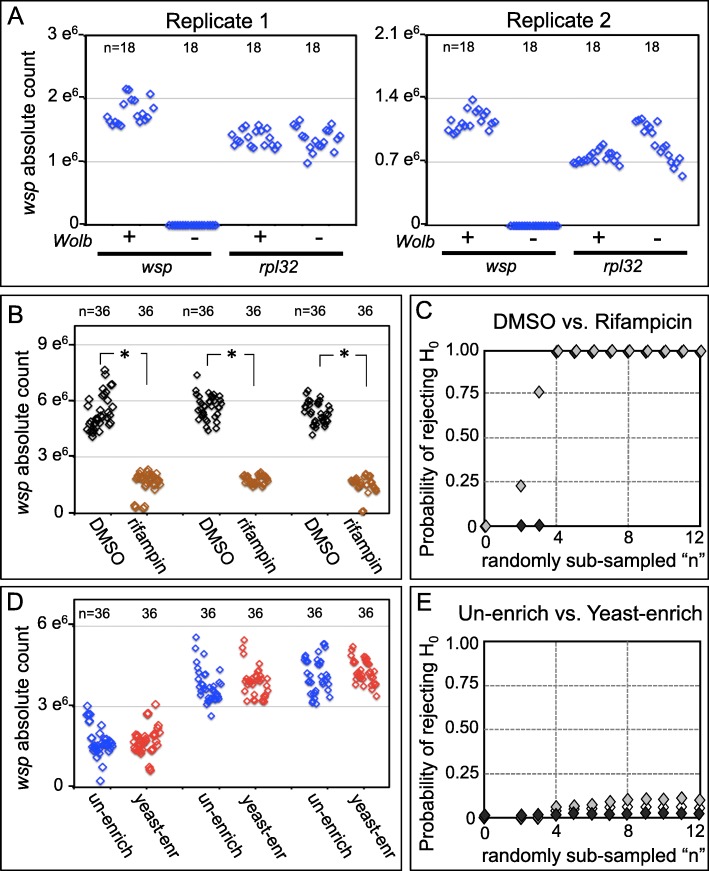
Absolute *wsp* abundance as indicated by real-time qPCR. Data from each sample/well represent 5 female flies. **a** Validation that bodywide *wsp* amplification by qPCR corresponds to *Wolbachia* infection. “n” represents 6 technical replicates from each of 3 sample tubes. **b** Test for bodywide *wsp* abundance changes within assayed timespan. Carrier DMSO and rifampicin conditions are shown. Data from 3 plate replicates are shown in pairs. n = 3 technical replicates from each of 12 wells. **c** Comparisons of randomly sub-sampled data from DMSO and rifampicin conditions, with alpha set at 0.01 n = 3–12 technical replicates (of total 36). Tests used per replicate: Plate 2 (grey): Welch’s T-test. Plate 3 (black): Mann-Whitney U. **d** Comparisons of bodywide *wsp* abundance in un-enriched versus yeast-enriched conditions. n = 3 technical replicates from 12 wells. **e** Comparing randomly sub-sampled data from un-enriched and yeast-enriched conditions, with alpha set at 0.01 n = 3–12 technical replicates (of total 36). Tests used per replicate: Plate 1 (white): Mann-Whitney U. Plate 2 (grey): Mann-Whitney U. Plate 3 (black): Welch’s T-test

Sample preparation was empirically optimized to maximize resolution of *wsp* abundance by absolute quantification. Use of detergent, proteinase K, specific temperatures, ethanol precipitation and a range of sample dilutions were systematically tested (Additional file 1: Figure S2). Specificity of template amplification was also tested by examining the abundance of *wsp* from fly stocks confirmed as *Wolbachia*(*−*) and *Wolbachia*(*+*) through staining and microbiome profiling (Figs. 2 and 3). Though real-time qPCR was able to amplify the fruit fly host gene *rpl32* from both *Wolbachia*(*−*) and *Wolbachia*(*+*) samples, the *wsp* gene was amplified in only the *Wolbachia*(*+*) samples (Fig. 6a) (Additional file 2: Table S7). The differential abundance of *wsp* signal in *Wolbachia*(*−*) and *Wolbachia*(*+*) flies confirms that *wsp* amplification by these qPCR methods specifically quantifies *Wolbachia* infection.

To determine whether body-wide qPCR can detect *Wolbachia* titer changes within the time frame of a feeding assay (Fig. 5), we tested whether rifampicin, an antibiotic drug previously shown to target *Wolbachia* [68–70], would reduce *wsp* abundance in *Wolbachia*(*+*) flies. Female flies were exposed to food supplemented with control DMSO or 100 μM DMSO-solubilized rifampicin in a 24-well plate format over 3 days (n = 7 females + 3 males per well, 12 wells per treatment condition) (Fig. 6b). Absolute *wsp* counts were then determined for 5 female flies per well. Rifampicin conditions exhibited 29% of the *wsp* abundance detected from DMSO control flies (*p* ≤ 0.001 as per statistical tests appropriate to each plate replicate) (Fig. 6b) (Additional file 2: Table S8 and Table S9). To determine whether adequate replication supported this conclusion, data subsets were selected at random and tested for significance. This analysis indicated that *wsp* absolute counts from 4 samples were sufficient to show a significant difference between rifampicin and control conditions (Fig. 6c) (Additional file 1: Figure S3) (Additional file 2: Table S9). These results, showing rifampicin suppression of bodywide *Wolbachia* titer, confirm that the optimized qPCR assay can detect bodywide titer changes in a timespan matching the germline titer assays reported above.

This validated qPCR method was next applied to test the effect of yeast-enriched host diets on body-wide *Wolbachia* titer. Female flies were fed un-enriched or yeast-enriched diets in a 24-well format for 3 days, then absolute *wsp* counts were measured via qPCR as above. This analysis found no significant difference in *wsp* abundance between un-enriched and yeast-enriched conditions (n = 12 wells per condition, 3 technical replicates per well) (Fig. 6d) (Additional file 2: Table S9 and Table S10). Sub-sampling analyses indicated less than 25% likelihood of significance with the α-value set conservatively at 0.01 (Fig. 6e) (Additional file 1: Figure S3) (Additional file 2: Table S9 and Table S10) [62, 71]. Overall the qPCR data indicate that, unlike control tests of rifampicin-fed flies, dietary yeast does not significantly affect body-wide *Wolbachia* titer. This suggests that the molecular mechanisms governing systemic *Wolbachia* loads are distinct from those that determine *Wolbachia* titer in maternal germline cells.

### Use of absolute qPCR shows that diet affects *Wolbachia* distribution within the body

The overt disparity across tissues raises a critical mechanistic question: How can germline cytology show *Wolbachia* sensitivity to dietary yeast if absolute counts of *Wolbachia* from whole-body samples do not? It is known that dietary yeast greatly increases ovary size [54, 55, 72]. Is ovarian *Wolbachia* depletion an artifact of ovary size, with the same number of bacteria spread out within a greater volume; or does *Wolbachia* depletion from oogenesis reflect an overall reduction in ovarian titer? To distinguish between these possibilities, we used the optimized methodology to quantify *Wolbachia* titer in whole flies and in dissected ovaries.

**Fig. 7:**
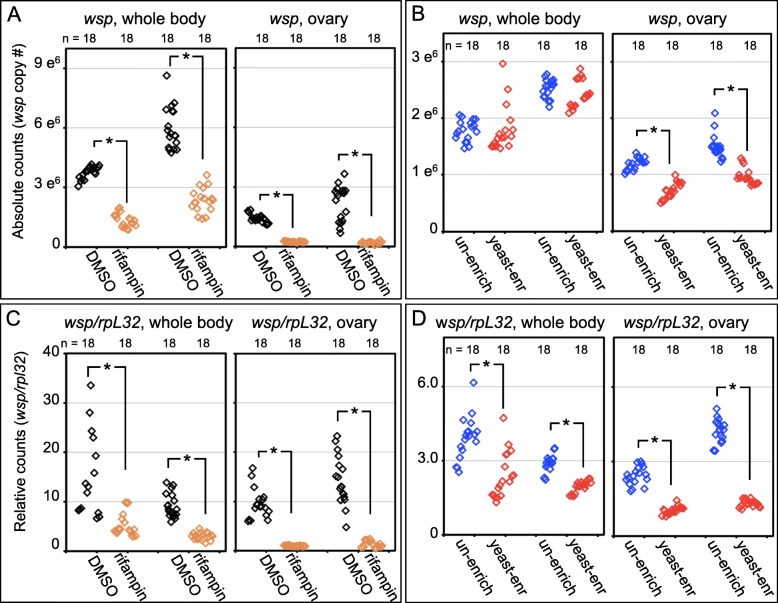
*wsp* abundance as indicated by real-time qPCR. Whole fly extracts and ovarian extracts are compared in each experiment. Panels show data from 2 independent plate replicates. “n” represents 6 technical replicates from 3 sample tubes. Data from each sample/well represent material from 5 female flies. **a** and **b** Absolute counts of *wsp* gene. *Wsp* abundance was compared in A) control DMSO vs. rifampicin treatment conditions, and B) un-enriched vs. yeast-enriched treatment conditions. **c** and **d** Relative counts, showing a ratio of *wsp/rpl32* abundance in **c** control DMSO vs. rifampicin treatment conditions, and **d** un-enriched vs. yeast-enriched treatment conditions. Statistical tests were applied as appropriate to each dataset, outlined in Additional file 2: Table S12 and S14

First, to confirm that absolute quantification yields results representative across whole body and ovarian samples, qPCR analyses were performed on rifampicin-treated samples. These results were consistent with the plate assay validation experiments performed above. Absolute quantification of *wsp* showed that rifampicin reduced whole body *Wolbachia* titers to 33–41% of the DMSO control (T-test, *p* < 0.001, n = 18) (Fig. 7a) (Additional file 2: Table S11 and Table S12). Rifampicin effects on ovarian *Wolbachia* titer were even more exaggerated, with rifampicin-treated ovaries showing 7–17% of control levels (Welch’s T-test, *p* < 0.001, n = 18) (Fig. 7a) (Additional file 2: Table S11 and Table S12). This demonstrates that ovarian samples can show qPCR-quantified *Wolbachia* titer responses to feeding treatments within the 3-day assay time period.

Next, to determine how ovarian *Wolbachia* titers respond to a nutrient-altered diet, we used qPCR to assay *Wolbachia* titer in whole bodies and ovarian samples from yeast-fed flies. Consistent with the data above, absolute quantification of *wsp* from whole body samples showed no significant difference between un-enriched and yeast-enriched food conditions (Various tests, *p* = 0.203–0.265, n = 18) (Fig. 7b) (Additional file 2: Table S13 and Table S14). Sub-sampling analyses confirmed that these conditions had only an 11–20% chance of satisfying a similar statistical significance, using the criterion of *p* < 0.01 (Additional file 1: Figure S4 and Additional file 2: Table S14). By contrast, absolute quantification of *wsp* from ovary samples exhibited a marked *Wolbachia* depletion in response to dietary yeast. Ovary tissues from yeast-fed flies exhibited 31–43% of the *Wolbachia* titer detected in the un-enriched controls (Various tests, *p* < 0.001, n = 18) (Fig. 7b) (Additional file 2: Table S13 and Table S14). Sub-sampling analyses reveals that this outcome is robust, as n = 6 would have been sufficient to satisfy the criterion of *p* < 0.01 (Additional file 1: Figure S4 and Additional file 2: Table S14). In summary, absolute counts indicate that *Wolbachia* titers are low in ovarian tissues of yeast-fed flies, even though whole body *Wolbachia* titers are stable. This suggests that low *Wolbachia* titers in late oogenesis reflect altered partitioning of *Wolbachia* between ovarian and somatic tissues.

### Relative qPCR yields misleading results from assessment of titer response to diet

Relative quantification using qPCR has been used to assess *Wolbachia* densities across diverse host systems [53, 73–77]. In this approach, *Wolbachia* titer is reported as a ratio of *wsp* versus a host gene, such as *rpl32.* This implicitly assumes that host DNA copy number remains stable across the conditions being tested. To test the applicability of relative quantification to germline colonization by *Wolbachia,* we estimated absolute copy number of *rpl32* in parallel with *wsp,* then calculated *wsp/rpl32* ratios from the absolute counts (Additional file 2: Table S11 and Table S13). In rifampicin control tests, results from relative quantification paralleled those from absolute counts. Here, *wsp/rpl32* ratios from rifampicin conditions were 30–36% of the ratios seen for control DMSO in whole body samples (Various tests, *p* < 0.001–0.043, n = 18) and 9–15% of control DMSO in ovarian samples (Various tests, *p* < 0.001–0.043, n = 18) (Fig. 7c) (Additional file 2: Table S11 and Table S12).

In contrast, under nutrient-altered conditions, relative *Wolbachia* titers were qualitatively different from our results with absolute counts. Interestingly, *wsp/rpl32* values were significantly lower in yeast-fed flies at the level of the whole body (T-test, *p* < 0.001, n = 18) as well as in ovarian tissues (Welch’s T-test, *p* < 0.001, n = 18) (Fig. 7d) (Additional file 2: Table S13 and Table S14). Sub-sampling analyses were consistent with this outcome, indicating 4–18 samples as sufficient to satisfy *p* < 0.01 in 98.5–100% of cases (Additional file 1: Figure S4 and Additional file 2: Table S14). Thus, outcomes using ratios (relative counts) suggest that dietary yeast suppresses bodywide *Wolbachia* titers, though absolute counts consistently show that bodywide titers are not yeast-sensitive. Ratios are misleading with respect to bodywide *Wolbachia* abundance because yeast-feeding induces a 1.5–1.9 fold median increase in absolute counts of *rpl32* in ovarian tissues, contradicting any assumption that host gene counts remain constant (Additional file 2: Table S13).

## Discussion

*Wolbachia* endosymbionts must overcome challenges similar to many bacterial pathogens when colonizing host cells. Direct observation of bacterial titer carried by host cells over time, and under different treatment conditions, is critical to inform the mechanisms of colonization. Technical limits on resolution of *Wolbachia* titer have impeded understanding of germline colonization to date. Empirical studies of germline *Wolbachia* titer have involved fluorescence intensity measurements from projections of the germarium and early oogenesis [22, 52], as well as selected focal planes from late oogenesis [35]. *Wolbachia* have also been quantified from 3-dimensional images of early to mid-oogenesis [31] and single focal planes from mid- and late oogenesis [49, 53, 54]. The methods presented here represent a major advance in providing clear *Wolbachia* resolution from germline stem cells through stage 10 egg chambers, representing 153 out of 162 h of oogenesis (Additional file 2: Table S15). In addition to enabling pursuit of mechanistic hypotheses, these methods enable systematic internal controls for consistency and accuracy of scoring across methods and cell types. Overall, this empirical resolution makes it possible to model germline colonization as an integrated process.

A general rationale for staining methods used to date has been that FISH and antibody stains for *Wolbachia* are necessary to avoid mis-attributing signal from other possible co-resident symbionts to *Wolbachia*. The ovary microbiome gene amplicon data corroborated nucleoid identities in DNA stained, *Wolbachia*(*+*) egg chambers as *Wolbachia.* Though ovary dissections were carefully performed to minimize contamination, our fruit flies were not raised in axenic conditions. Low-level microbial background signal is diverse and variable, as reflected by detection of over 200 non-*Wolbachia* genera in all samples analyzed, regardless of infection status. We cannot rule out the possibility that extremely low-level background *Wolbachia* are carried by flies that were otherwise indicated as uninfected by standard qPCR, quantitative qPCR and cytological staining. However, due to re-use of dissection equipment, it is possible that trace amounts of *Wolbachia* DNA detected in uninfected samples by 16S rRNA gene profiling represent basal contamination of dissecting equipment. Furthermore, neither *Spiroplasma*, nor *Buchnera,* nor dozens of other known insect endosymbionts [12] were identified by the ovary microbiome analyses. This confirms that punctate nucleoids observed in *Wolbachia-*infected *D. melanogaster* ovary tissues represent *Wolbachia,* and can be analyzed with confidence in that regard. To our knowledge, 16S microbiome analyses have not previously been used to confirm nucleoid identity in insect germline models of endosymbiosis. Inclusion of this approach as a control in future studies is now possible due to increased accessibility and affordability of such analyses.

A major outcome from this study was that absolute counts showed equivalent *Wolbachia* titers across nutrient-altered diets, whereas relative quantification did not. The basis for this effect was an increase in baseline host *rpl32* levels in yeast-fed flies. This makes sense considering the biology of reproduction. Most homometabolous insects, like *Drosophila,* have meroistic, polytrophic ovaries, in which each oocyte has a dedicated set of 15 nurse cells that load the oocyte with all content needed for embryogenesis [78]. To support mass production, *Drosophila* nurse cell nuclei endoreplicate their DNA. This yields ploidy on the order of 2000+ for any given nurse cell [79], and intrinsically increases *rpl32* copy number per host. As such, any treatment that affects nurse cell ploidy or ovary productivity will certainly also affect *rpl32* abundance. The absolute-count methodology we present can be generalized to any mutant background or drug treatment condition in future studies. As there is no way to anticipate *rpl32* responses to new experimental conditions, absolute quantification approaches are important to acquire reliable data to test models of tissue-specific effects in complex biological systems.

**Fig. 8:**
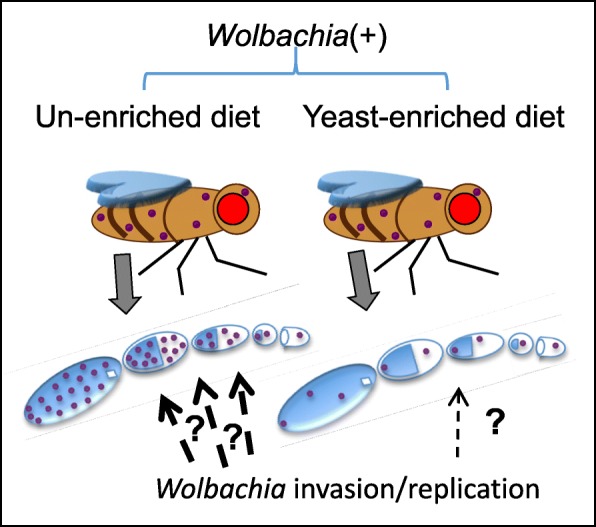
Model for *D. melanogaster* germline colonization by *Wolbachia.* Female fruit flies and corresponding ovarioles are shown. *Wolbachia* infection is indicated by purple dots. *Wolbachia* titers carried bodywide and in early oogenesis did not show any consistent response to host diet. However, *Wolbachia* titers from late oogenesis are markedly lower in yeast-enriched conditions than on un-enriched food. This is consistent with possible developmental regulation of *Wolbachia* invasion and/or replication in maternal germline cells

Our quantitative cytological analyses can detect developmental sensitivity to host nutrition. Germline stem cell titers demonstrated modest sensitivity to host dietary yeast in this study. This may represent a dilution effect caused by increased GSC division rates, directed by yeast-driven insulin signaling [55, 72] (Fig. 8). However, depletion of *Wolbachia* from GSCs is not ultimately responsible for late-stage titer depletion in yeast-fed flies. The uniform titer obtained in egg chambers by stage 4 invokes an internal titer correction mechanism of unknown origin. The stability of whole body titer, despite a decrease in ovarian titers, further suggests that yeast-driven insulin signaling triggers redistribution of *Wolbachia* within the body (Fig. 8). This is in agreement with published findings that ovarectomized females exhibit higher somatic *Wolbachia* titers in yeast-enriched conditions [53]. One interpretation is that insulin suppresses invasion of late stage germline cells by somatic *Wolbachia.* An alternative possibility is that insulin favors somatic replication while suppressing *Wolbachia* replication in late oogenesis. A current limitation of this assay is that it does not inform replication or binary fission rates. We are currently pursuing the effects of insulin on germline colonization and *Wolbachia* binary fission as part of a separate study.

The methodology presented here can be adapted to many research questions. Adult-only feeding experiments were done to specifically address how food affects colonization of existing, healthy maternal germline cells. Field literature has reported that egg development occurs over an approximately 6-day period (Additional file 2: Table S15) [80–82]. With this knowledge, future studies can adapt preparation conditions to calibrate the developmental window of interest, using narrower treatment times to detect more specific developmental impacts. Alternatively, treatment timelines can be expanded to assess more cumulative effects across life cycle stages. It will be possible to further explore other processes implicated in germline titer control, such as *Wolbachia* impacts on actin polymerization [21, 52, 83, 84] and vesicle trafficking pathways [4, 5, 85–89] relevant to oogenesis. Our methodology will translate readily across other *Wolbachia/*host combinations, and may be adapted to other insect tissues or possibly endosymbiont/host models. A limitation of our approach is that alternative staining methods like FISH would be required to study multiply infected hosts.

Going forward, it is also important to consider that DNA extraction and amplification may vary substantially across host taxa, host tissues and endosymbiont types [90, 91]. Inclusion of control DNA, spiked into pre- and post-extraction samples, will be needed for accurate data interpretation in comparative analyses [92]. The absence of such controls is a limitation of our current study. For example, the data presented in Fig. 7, when extrapolated back to source material, would appear to imply that flies raised on control food carry an average of 43 million *Wolbachia*, with 27 million bacteria resident in ovarian tissues. Additional controls would be needed to confirm such an interpretation, however. Addition of control DNA to pre-extracted samples would be needed to confirm the consistency of DNA retention across sample types during DNA extraction. Adding known amounts of control DNA post-extraction, and amplifying that by qPCR, would further indicate whether qPCR efficacy differs across sample types [90, 91]. Use of spiked-in DNA controls in future qPCR analyses will support pursuit of testable models, based upon robust findings from diverse experimental systems [90, 91].

This experimental methodology is presented with an atypical approach to statistical analysis. We outline a methodology for selecting both appropriate statistical tests and relevant sample sizes. An α-value of 0.05 is considered standard in many disciplines as sufficient to reject the null hypothesis and conclude that there is a statistically meaningful difference between comparison groups [93, 94]. However, sub-sampling the data to identify the “n” required for significance at α = 0.01 further informs the scale of the differences observed between conditions, such as between GSC and stage 10 titer responses to yeast-enriched diets. Direct review of the empirical data is also important. In this study, titer trends were evident in stage 10 titer data from all controls, as well as in GSCs from the un-enriched controls. Time-correlated data implicate some form of “non-demonic intrusion” (i.e. unknown secondary causes of differences observed in an experiment) as a source of *Wolbachia* titer variation in control fly populations [85, 95]. Investing in analyses of control conditions also identifies potential false positives, as implicated for GSC titers in un-enriched conditions. From this, we conclude that a minimum sample size of 15 and an operational α-value of 0.01 will be useful standards in this regard. This will help ensure that interpretations are based on reliable and repeatable effects of host processes on *Wolbachia* titers and avoid artifacts due to spurious statistical findings.

## Conclusions

Clear resolution of bacterial titer carried by eukaryotic cells is critical to understanding mechanisms involved in host colonization. The methodology presented here enables accurate, reproducible and rigorous measurement of endosymbiotic *Wolbachia* bacteria across maternal germline development. The methods demonstrated that *Wolbachia* titer is distinctly nutrient-sensitive in late stages of oogenesis, consistent with bacterial redistribution within the insect host. Optimized titer assessments, provided by the molecular, cytological, and statistical approaches detailed here for the well-described *Drosophila melanogaster* model system, will advance understanding the complex mechanisms of endosymbiosis and vertical transmission.

## Methods

### Fly stocks & food preparation

Fly stocks and food preparation were as described elsewhere [54, 56]. Ovary preparations were done using flies of genotype *w; Sp/Cyo; Sb/Tm6B* carrying the *w*Mel *Wolbachia* strain [56]. Uninfected control flies of this same genotype represent the original parental strain, prior to addition of *w*Mel. Newly eclosed, adult flies were aged for 5 days in a controlled, 25 °C environment. Twenty females and 5 males were initially placed into each vial, with the first 2 days of rearing done on standard food, followed by transfer to fresh food containers. For the next 3 days of rearing, adult flies were exposed to appropriate food conditions for the experiment. Food was prepared into batches, then dispensed into individual vials or plate wells, and used immediately following cooling to ensure consistency of feeding. For the initial set of cytological experiments using control food, flies were kept in vials with standard fly food for 3 days. For diet-related experiments, each vial of “yeast-enriched” food represented 1.5 mL heat-inactivated yeast paste stirred into 3.5 mL melted standard food, stirred until homogeneous and smooth. The “un-enriched” food used in parallel represents 3.5 mL melted standard food mixed with 1.5 mL water [54]. The nutritional profile associated with these foods was determined by Medallion labs (Minneapolis, MN) (Additional file 3: S7).

Plate assay experiments contained 1 mL of fly food per well. For control antibiotic experiments in the plate assay format, 200 μL of DMSO or 10 mM rifampicin-DMSO stock solution were stirred into 20 mL of melted standard food and dispensed into plate wells. This resulted in a final DMSO concentration of 1%, and a 100 μM dose for the rifampicin condition.

### Microbial 16S rRNA gene sequencing of ovarian tissue

Both uninfected and *w*Mel-infected *D. melanogaster* flies of the genotype *w; Sp/Cyo; Sb/TM6B* were reared on normal food and prepared as described above. Three pools of 20 ovaries of each type were dissected in 0.1 M Tris HCl, 0.1 M EDTA, rinsed twice with fresh buffer, and homogenized in 50 μl lysis buffer from DNeasy (Qiagen) Blood and Tissue Extraction Kit. Total DNA was extracted according to manufacturer’s instructions, estimated by fluorimetry on a Qubit 2.0 (Life Technologies), precipitated and dried. All samples of more than 50 ng total were sent to Omega Bioservices (Norcross, GA) for Next-Gen, PCR-targeted sequencing. Briefly, primers covering the V1-V3 regions of bacterial 16S rRNA gene, 27F (5′- AGAGTTTGATCCTGGCTCAG) and 534R (5′-ATTACCGCGGCTGCTGG), were used to amplify and sequence on an Illumina MiSeq with V3 chemistry. Our target was total of 50,000 reads per sample, attainable from 25 cycles of PCR for *Wolbachia*(+) samples, though 2 of 3 *Wolbachia*(−) samples required 30 cycles to amplify sufficient signal for sequencing. Result analyses were performed via Illumina’s BaseSpace 16S rRNA application module, using the Illumina-curated version of May 2013 Greengenes taxonomic database in parallel with the Ribosomal Database Project for taxonomic classification of constituent microbial populations.

### Tissue staining and imaging

Staining procedures were modified from [57]. Ovaries were dissected from 5 day old flies in phosphate-buffered saline (PBS), then fixed for 25 min in a mixture of 400 μl heptane, 112.5 μl of 32% EM-grade paraformaldehyde (Electron Microscopy Sciences, cat # 15714) and 387.5 μl MEH buffer (2 mM Mg2SO4, 1 mM EGTA, 0.1 M Hepes pH 6.9). Tissues were rinsed 3X with PBS-0.1% Triton, washed 2X for 10 min with PBS-0.3% Triton, and rinsed 3X in PBS. Ovaries were incubated overnight at room temperature in 10 mg/mL RNAse A (Sigma Cat # R5503). Tissues were then washed in fresh PBS-0.1% Triton every 15 min for a total of 2 h and resuspended in 70% glycerol containing 0.015 mg/mL propidium iodide. After 2 days of incubation in the dark, the ovaries were slide-mounted, separated into ovarioles and sealed with a coverslip.

Ovarioles were imaged by laser-scanning confocal microscopy. An Olympus FV1200 confocal microscope was used at 60X magnification. Images were acquired from top to bottom of each sample at 1.5 μm Z-intervals. Similar intensity settings were applied to all egg chambers imaged in each replicate. Germaria and stage 4 egg chambers were visualized at 3X zoom. Stage 10 egg chambers were imaged at 1.5X zoom. Stage 10 oocytes and nurse cells were acquired separately due to size, with the same settings for comparability. About 20 flies were used per condition for each round of staining, resulting in approximately 20 candidate ovarioles per slide. Of those, approximately 2 ovarioles contained image-able material for all timepoints of interest: GSCs, stage 4, and stage 10 egg chambers. In terms of overall throughput, 300 flies, processed in 15 staining rounds, enabled imaging of 30 ovarioles with desired staging. Uninfected ovarian tissues were stained and imaged as a control.

### Quantification of *Wolbachia* from germline cell images

To quantify *Wolbachia* titer in early oogenesis, relevant focal planes were analyzed from the distal tip of each ovariole. Cells in direct contact with anterior, terminal filament cells in the germarium were identified as putative GCSs [26]. For single GSC counts, *Wolbachia* were manually scored in all focal planes of the distal-most cell. For *Wolbachia* counts in GSC clusters, all cells in contact with the terminal filament were analyzed. Germaria have been reported to typically carry 2–3 GSCs [80, 96]. Our GSC selection criteria identified 2–4 putative GSCs per ovariole. Therefore, it is possible that a subset of titer data associated with GSC clusters is attributable to GSC daughter cells. Manual quantification of *Wolbachia* was also carried out in stage 4 germline cyst cells. Germline cells were differentiated from somatic follicle cells by size and morphology. Though the entirety of each egg chamber was imaged, *Wolbachia* were manually scored for germline cells in appropriate focal planes.

To quantify *Wolbachia* titer in stage 10 germline cysts, a semi-automated approach was used. As egg chambers at this stage are roughly football shaped, the focal plane showing the largest sample width represents the Z-center of the egg chamber. Focal planes down to half the Z-depth of the egg chamber yielded sufficient resolution for analysis and were thus pursued. Images from each focal plane were manually processed in Adobe Photoshop to remove the follicle cells and any extraneous host DNA staining signal, unrelated to germline *Wolbachia* nucleoids [49]. After thresholding the images to eliminate background noise, the images were inverted and *Wolbachia* titer quantified by the Analyze Particles feature in Fiji (NIH Image J) software available at https://imagej.net/. *Wolbachia* counts from all quantified focal planes were doubled to approximate *Wolbachia* titer for the entire Z-depth of stage 10 germline cysts.

Redundancy of puncta across confocal imaging planes was assessed in paired sets of images selected from random Z-heights of 15 stage 10 oocytes. Images were derived from the low-titer, yeast-enriched condition to reduce the likelihood of misinterpreting neighbor *Wolbachia* across multiple focal planes as a single microbe. Signal overlap of 2 pixels or more suggested approximately 5% redundancy of *Wolbachia* counts between focal planes (Additional file 2: Table S16).

### DNA extraction and bodywide qPCR of *Wolbachia* titer

For total bodywide counts from each sample, a group of 5 female flies was homogenized together in 200 μl of buffer containing 10 mM Tris HCl (pH 8.0), 1 mM EDTA and 25 mM NaCl, with or without 1% SDS. Additionally, samples were processed with or without the addition of 2 μl of 20 mg/ml of proteinase K, followed by incubation at either 56 °C or 70 °C. After incubation for 1 h, samples treated with proteinase K were inactivated by heating the samples at 95 °C for 3 min. Samples were then centrifuged at 14,000 rpm for 15 min at 4 °C. Avoiding the pellet, 100 μl of supernatant was collected and DNA was either used directly for qPCR, diluted in TE, or was concentrated by ethanol precipitation. For precipitation, 1/10 volume of 3 M Na-acetate and 250 μl of absolute ethanol was added to 100 μl of the supernatant. Samples were mixed gently and kept at − 20 °C for > 2 h, then centrifuged at 14,000 rpm for 15 min at 4 °C. Resulting pellets were washed with 500 μl of 70% ethanol, and re-centrifuged at 14,000 rpm for 15 min at 4 °C. The DNA pellet was air dried and re-suspended in 100 μl of TE buffer. DNA samples were then used directly, or serially diluted for qPCR.

Absolute quantification of *Wolbachia* was carried out using reference plasmid standards that carry a 160 bp PCR-amplified fragment of the *Wolbachia* surface protein *(wsp*) gene [56]. Real-time PCR was carried out on a Bio-Rad CFX96 Connect Optics Module Real-Time System and absolute copy numbers for *Wolbachia* were obtained by comparing threshold cycle (*C*_*t*_) values with a standard curve generated from the plasmid standard, as in [56]. An additional plasmid standard was also prepared in parallel, from *D. melanogaster ribosomal protein L32* (*rpl32*) to standardize sample loading in *Wolbachia*(−) samples. These plasmids were prepared by cloning a 194 bp fragment of *rpl32* using forward (5′-CCGCTTCAAGGGACAGTATC) and reverse (5′- CAATCTCCTTGCGCTTCTTG) primers.

### Statistical analysis

All primary data collected in this study were matched with appropriate statistical analyses, as per a decision tree outlined in (Additional file 1: Figure S5). Data were analyzed for consistency with a normal distribution using the Shapiro-Wilk test, and for homogeneity of variances using Levene’s test [97–99]. For normal data, distributions showing homogenous variances were compared by T-test. Distributions with unequal variances were compared by Welch’s T-test [61, 100]. For non-normal data, distributions with homogeneous variances were compared using the Mann-Whitney U test [61, 62]. For non-normal distributions with unequal variances, significance was estimated using randomization based T-tests with bootstrapping, as recommended by field literature [62, 96, 98–100]. For non-parametric comparisons of data across 3 developmental stages, a Kruskal-Wallis ANOVA was performed. The IBM SPSS v.23 analysis package was used for all statistical tests performed in this study [101].

We were unsure how many samples would suffice to reliably detect differences in *Wolbachia* titer across different conditions. Having collected 15–36 samples per subject group, we conducted power analysis to determine the smallest number of samples that would likely be needed to reveal a significant difference, with a mind toward achieving greater economy of effort in future projects. To assess the power of different sample sizes, we used a sub-sampling procedure programmed by Dr. Philip K. Stoddard in MATLAB™ (Mathworks, Natick MA) that sampled randomly with replacement from *Wolbachia* titer datasets being compared (Additional file 4: S8). The script (Wol_power) tested for titer differences between the control and treatment conditions for each sub-sample set. Sub-samples ranged from 2 to 35 data points, with 10,000 sample iterations per sample size. Significance was assessed in accordance with the normality of data being analyzed, using T-tests (ttest2, with variance settings adjusted to match the data) and Mann-Whitney U (ranksum) [102, 103]. The α-value was set at 0.01, two-tailed. A summary graphic for each analysis indicates the proportion of significant results obtained for each sub-sample size. This power analysis of reduced datasets informs the level of certainty associated with observed *Wolbachia* titer differences.

## Additional files

Additional file 1: **Figure S1.** *Wolbachia* quantification through a semi-automated approach. **Figure S2.** Optimization of sample prep for absolute, real-time qPCR. **Figure S3.** Comparing randomly sub-sampled data for *wsp* absolute counts from different experimental conditions. **Figure S4.** Comparisons of randomly sub-sampled data for whole body versus ovarian samples under different dietary conditions. **Figure S5.** Selection of statistical methods for pairwise data comparisons. (PPTX 871 kb)

Additional file 2: **Table S1.** Highest abundance 16S metagenomic reads from *D. melanogaster* ovary samples. **Table S2.** 3-dimensional *Wolbachia* titer counts from maternal germline cells of *D. melanogaster*. **Table S3.** Statistical analyses of cytological *Wolbachia* titer data. **Table S4.** Quantifying average *Wolbachia* titer per cell in GSC clusters. **Table S5.** Comparing approaches for cytological *Wolbachia* quantification. **Table S6.** 3-dimensional *Wolbachia* counts from maternal germline cells, in response to different host diets. **Table S7.** Absolute quantification of host and *Wolbachia* DNA copy number by real-time qPCR. **Table S8.** Absolute quantification of *Wolbachia* DNA copy number, following host exposure to antibiotics. **Table S9.** Statistical analyses of qPCR-based *Wolbachia* titer data. **Table S10.** Absolute quantification of *Wolbachia* DNA copy number, following host exposure to nutrient-altered food. **Table S11.** Quantification of *Wolbachia* and host DNA copy number from whole body and ovarian samples, in response to Rifampicin treatment. **Table S12.** Statistical analyses of Wsp abundance from whole body and ovary tissues, in response to Rifampicin treatment. **Table S13.** Quantification of *Wolbachia* and host DNA copy number from whole body and ovarian samples, in response to nutrient-altered food. **Table S14.** Statistical analyses of Wsp abundance from whole body and ovary tissues, in response to nutrient-altered food. **Table S15.** Published timelines of germline development in *D. melanogaster*. **Table S16.** Test of signal overlap between imaging planes. (XLSX 83 kb)

Additional file 3: **S1.** *Wolbachia*(−) ovary microbiome, Replicate 1. **S2.** *Wolbachia*(−) ovary microbiome, Replicate 2. **S3.** *Wolbachia*(−) ovary microbiome, Replicate 3. **S4.** *Wolbachia*(+) ovary microbiome, Replicate 1. **S5.** *Wolbachia*(+) ovary microbiome, Replicate 2. **S6.** *Wolbachia*(+) ovary microbiome, Replicate 3. **S7.** Nutritional content of the food sources used in this study (XLSX 162 kb)

Additional file 4: Christensen et. al. 2018 submission to BMC Microbiology (DOCX 54 kb)

## Data Availability

All data generated or analyzed during this study are included in this published article and its supplementary information files.

## Abbreviations

GSC: Germline stem cell
IPC: Insulin producing cell
*rpl32*: *Ribosomal protein L32* gene
*w*Mel: *Wolbachia* strain endogenous to *D. melanogaster*
*wsp*: *Wolbachia surface protein* gene
