# Supplementary material for: Quantitative methods for assessing local and bodywide contributions to Wolbachia titer in maternal germline cells of Drosophila
Source: BMC Microbiol. 2019 Sep 3;19:206. doi: 10.1186/s12866-019-1579-3 (PMC6724367; doi:10.1186/s12866-019-1579-3)
Supplement: Supplementary file 1 — Figure S1. Wolbachia quantification through a semi-automated approach. Figure S2. Optimization of sample prep for absolute, real-time qPCR. Figure S3. Comparing randomly sub-sampled data for wsp absolute counts from different experimental conditions. Figure S4. Comparisons of randomly sub-sampled data for whole body versus ovarian samples under different dietary conditions. Figure S5. Selection of statistical methods for pairwise data comparisons. (PPTX 871 kb) [file 12866_2019_1579_MOESM1_ESM.pptx]

## Slide 1
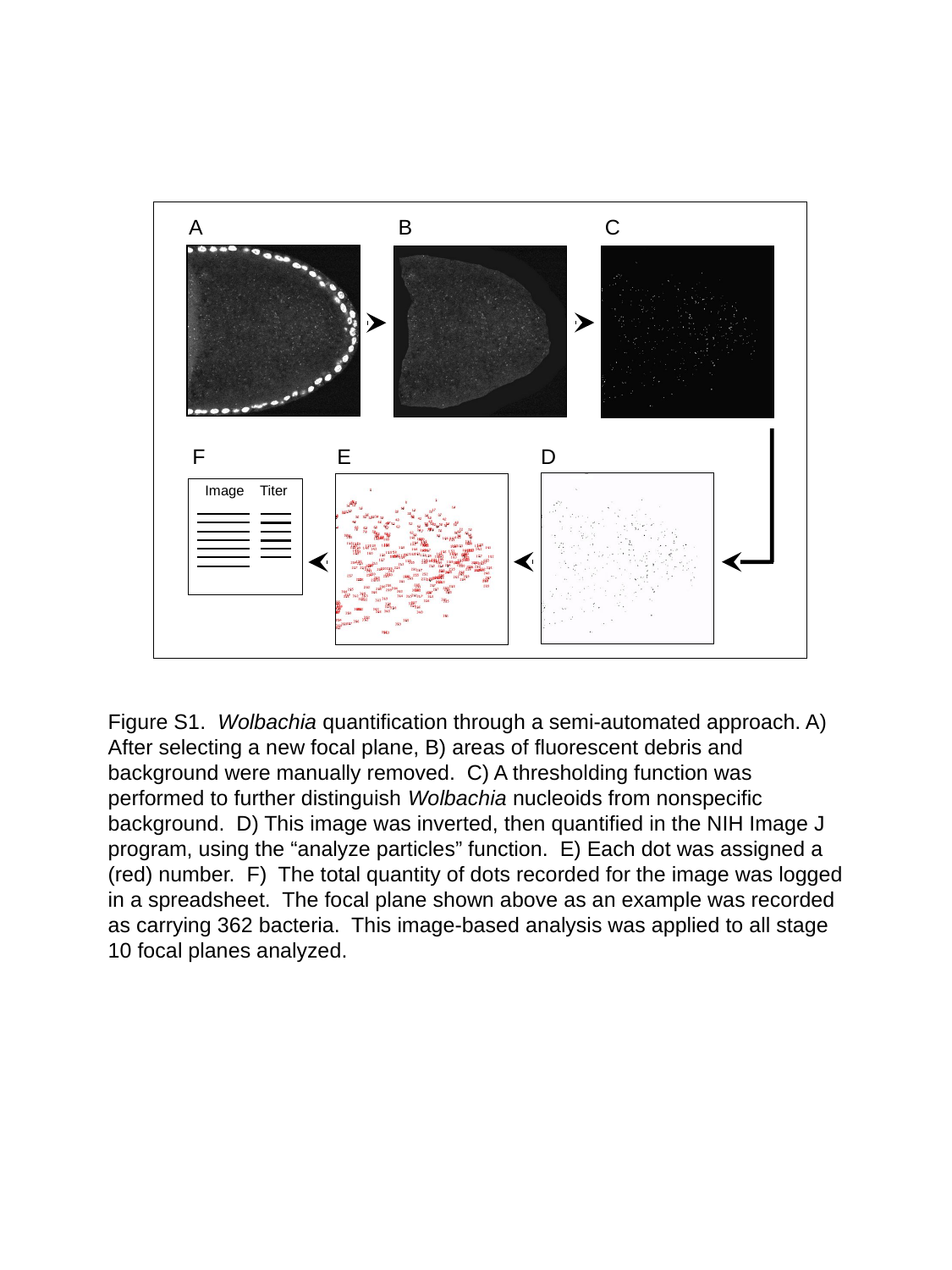

A 	 B		 C
F 	 E	 D
Image Titer
Figure S1. Wolbachia quantification through a semi-automated approach. A) After selecting a new focal plane, B) areas of fluorescent debris and background were manually removed. C) A thresholding function was performed to further distinguish Wolbachia nucleoids from nonspecific background. D) This image was inverted, then quantified in the NIH Image J program, using the “analyze particles” function. E) Each dot was assigned a (red) number. F) The total quantity of dots recorded for the image was logged in a spreadsheet. The focal plane shown above as an example was recorded as carrying 362 bacteria. This image-based analysis was applied to all stage 10 focal planes analyzed.

## Slide 2
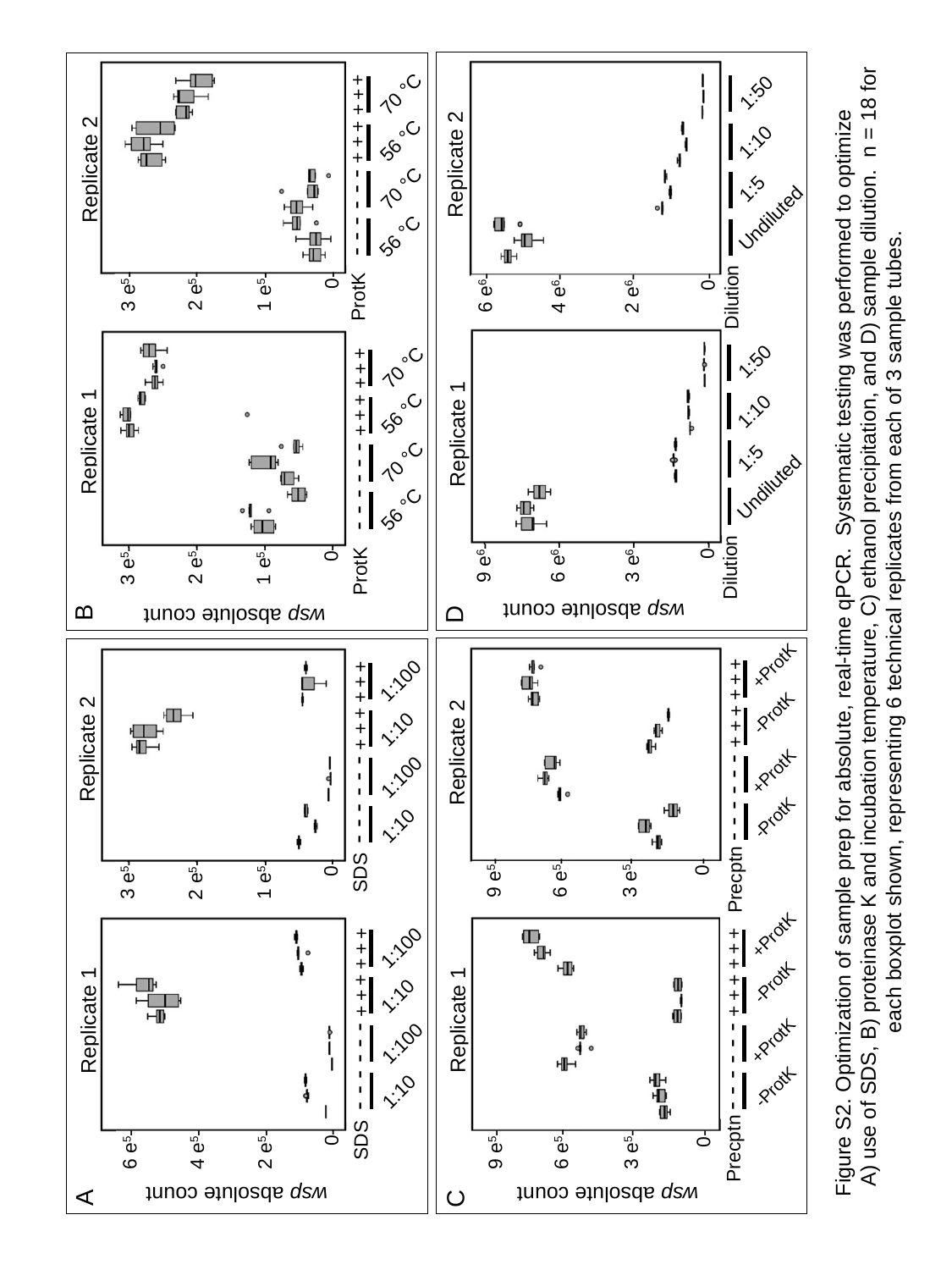

Replicate 2
6 e6-
4 e6-
2 e6-
0-
Dilution
1:5
Undiluted
1:50
1:10
3 e5-
2 e5-
1 e5-
0-
- - - - - -
+
+
+
+
+
+
56 °C
70 °C
ProtK
56 °C
70 °C
Replicate 2
Replicate 1
wsp absolute count
Dilution
1:5
Undiluted
1:50
1:10
3 e5-
2 e5-
1 e5-
0-
- - - - - -
+
+
+
+
+
+
56 °C
70 °C
ProtK
56 °C
70 °C
wsp absolute count
Replicate 1
9 e6-
6 e6-
3 e6-
0-
Figure S2. Optimization of sample prep for absolute, real-time qPCR. Systematic testing was performed to optimize A) use of SDS, B) proteinase K and incubation temperature, C) ethanol precipitation, and D) sample dilution. n = 18 for each boxplot shown, representing 6 technical replicates from each of 3 sample tubes.
B
D
9 e5-
6 e5-
3 e5-
0-
- - - - - -
+
+
+
+
+
+
+ProtK
-ProtK
-ProtK
+ProtK
Precptn
Replicate 2
3 e5-
2 e5-
1 e5-
0-
- - - - - -
+
+
+
+
+
+
1:10
1:10
1:100
1:100
SDS
Replicate 2
Replicate 1
6 e5-
4 e5-
2 e5-
0-
- - - - - -
+
+
+
+
+
+
1:10
1:10
1:100
1:100
SDS
9 e5-
6 e5-
3 e5-
0-
- - - - - -
+
+
+
+
+
+
+ProtK
-ProtK
+ProtK
-ProtK
Precptn
Replicate 1
A
C
wsp absolute count
wsp absolute count

## Slide 3
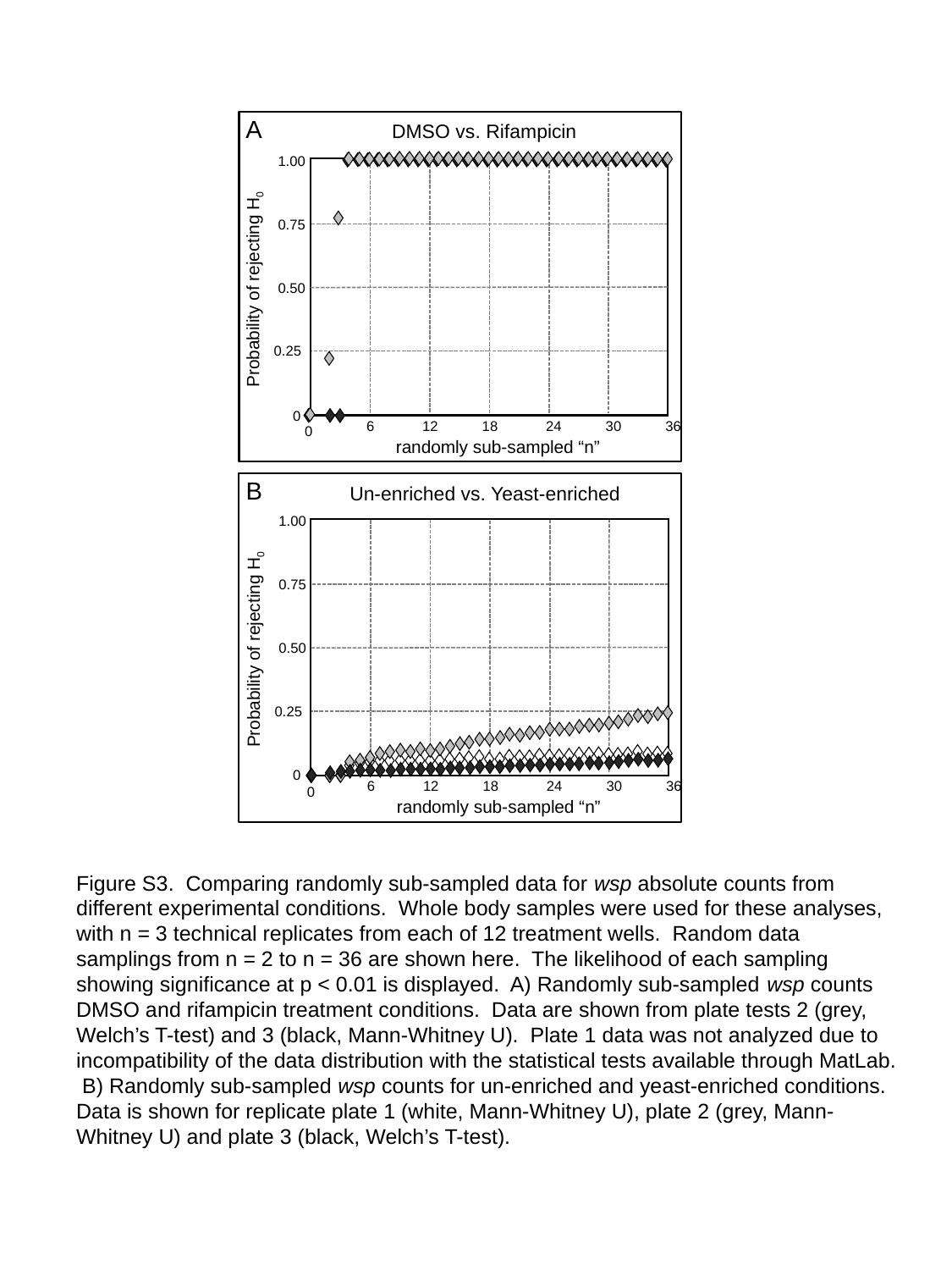

A
DMSO vs. Rifampicin
1.00
0.75
0.50
0.25
Probability of rejecting H0
0
 12 18 24 30 36
0
randomly sub-sampled “n”
B
Un-enriched vs. Yeast-enriched
1.00
0.75
0.50
0.25
Probability of rejecting H0
0
 12 18 24 30 36
0
randomly sub-sampled “n”
Figure S3. Comparing randomly sub-sampled data for wsp absolute counts from different experimental conditions. Whole body samples were used for these analyses, with n = 3 technical replicates from each of 12 treatment wells. Random data samplings from n = 2 to n = 36 are shown here. The likelihood of each sampling showing significance at p < 0.01 is displayed. A) Randomly sub-sampled wsp counts DMSO and rifampicin treatment conditions. Data are shown from plate tests 2 (grey, Welch’s T-test) and 3 (black, Mann-Whitney U). Plate 1 data was not analyzed due to incompatibility of the data distribution with the statistical tests available through MatLab. B) Randomly sub-sampled wsp counts for un-enriched and yeast-enriched conditions. Data is shown for replicate plate 1 (white, Mann-Whitney U), plate 2 (grey, Mann-Whitney U) and plate 3 (black, Welch’s T-test).

## Slide 4
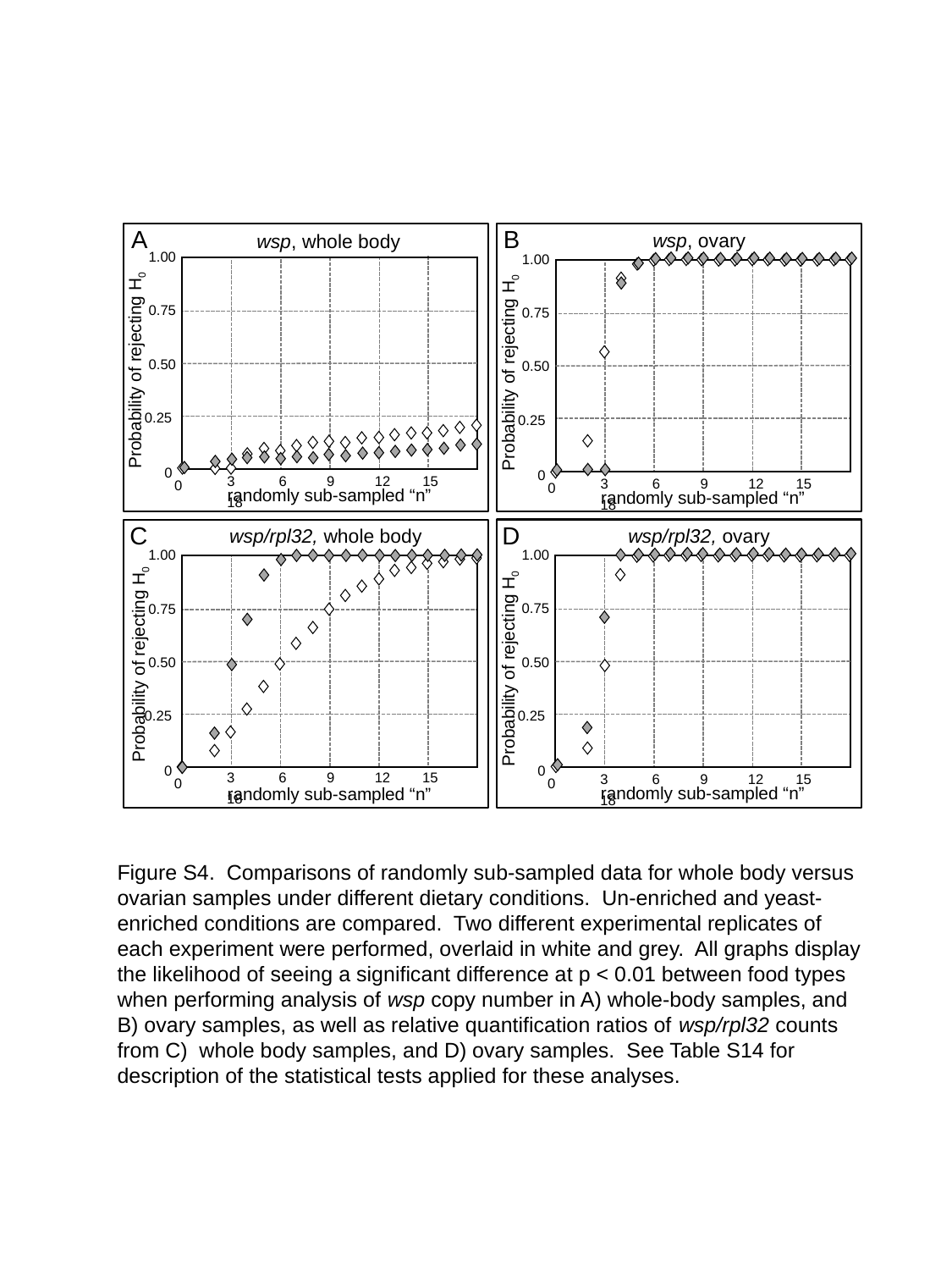

B
wsp, ovary
1.00
0.75
0.50
0.25
Probability of rejecting H0
0
3 6 9 12 15 18
0
randomly sub-sampled “n”
A
wsp, whole body
1.00
0.75
0.50
0.25
Probability of rejecting H0
0
3 6 9 12 15 18
0
randomly sub-sampled “n”
D
wsp/rpl32, ovary
1.00
0.75
0.50
0.25
Probability of rejecting H0
0
3 6 9 12 15 18
0
randomly sub-sampled “n”
C
wsp/rpl32, whole body
1.00
0.75
0.50
0.25
Probability of rejecting H0
0
3 6 9 12 15 18
0
randomly sub-sampled “n”
Figure S4. Comparisons of randomly sub-sampled data for whole body versus ovarian samples under different dietary conditions. Un-enriched and yeast-enriched conditions are compared. Two different experimental replicates of each experiment were performed, overlaid in white and grey. All graphs display the likelihood of seeing a significant difference at p < 0.01 between food types when performing analysis of wsp copy number in A) whole-body samples, and B) ovary samples, as well as relative quantification ratios of wsp/rpl32 counts from C) whole body samples, and D) ovary samples. See Table S14 for description of the statistical tests applied for these analyses.

## Slide 5
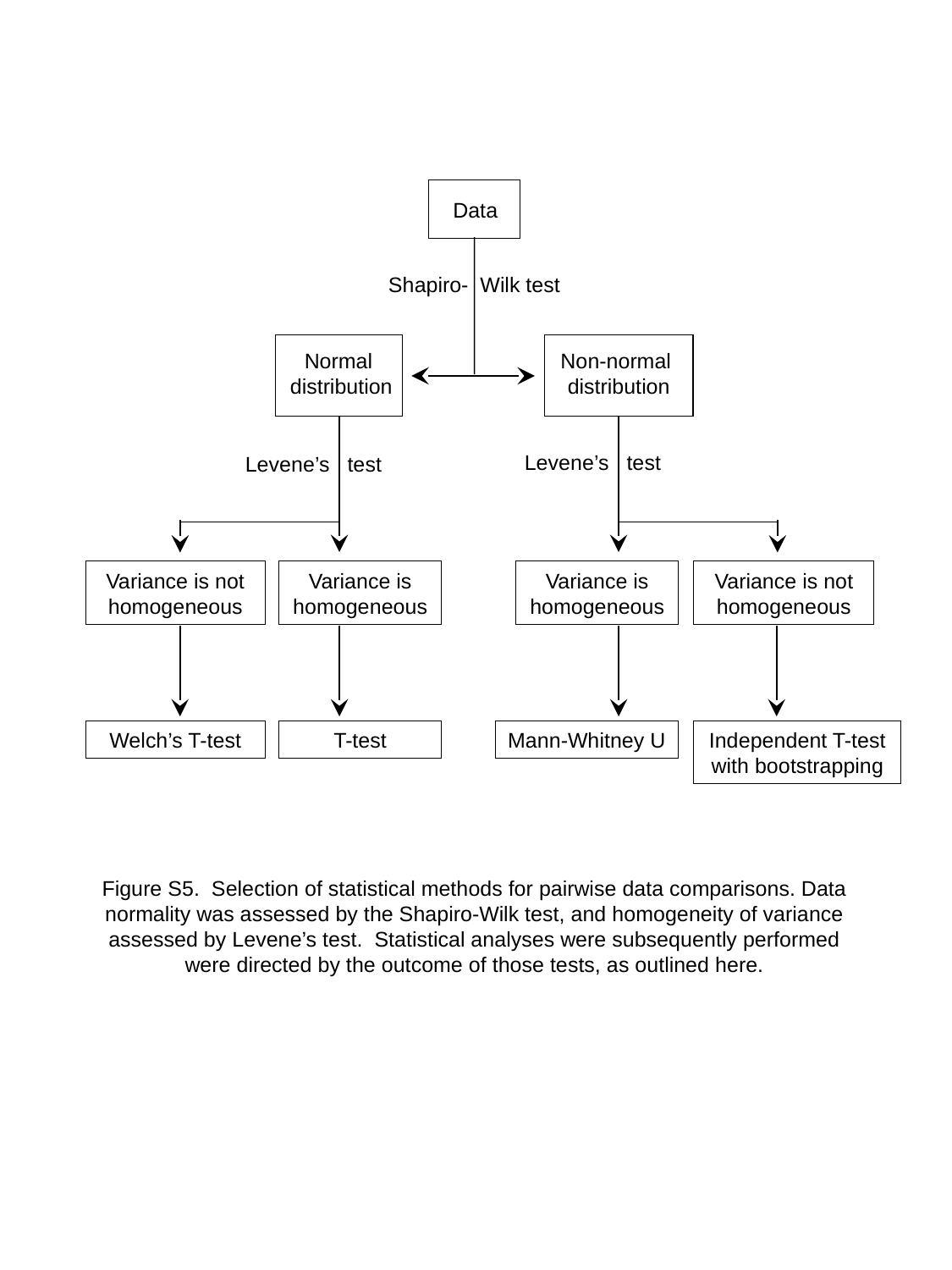

Data
Shapiro- Wilk test
Non-normal
distribution
Normal
distribution
Levene’s test
Levene’s test
Variance is not homogeneous
Variance is homogeneous
Variance is homogeneous
Variance is not homogeneous
Welch’s T-test
T-test
Mann-Whitney U
Independent T-test with bootstrapping
Figure S5. Selection of statistical methods for pairwise data comparisons. Data normality was assessed by the Shapiro-Wilk test, and homogeneity of variance assessed by Levene’s test. Statistical analyses were subsequently performed were directed by the outcome of those tests, as outlined here.
