# Supplementary material for: Quantitative methods for assessing local and bodywide contributions to Wolbachia titer in maternal germline cells of Drosophila
Source: BMC Microbiol. 2019 Sep 3;19:206. doi: 10.1186/s12866-019-1579-3 (PMC6724367; doi:10.1186/s12866-019-1579-3)
Supplement: Supplementary file 4 — Christensen et. al. 2018 submission to BMC Microbiology (DOCX 54 kb) [file 12866_2019_1579_MOESM4_ESM.docx]

**Christensen et. al. 2018 submission to BMC Microbiology**

**Additional File S7**

This document provides the MATLAB code necessary to run the Wol_power script developed for this study. Three separate coding files are required, with the sem.m and Lauraplot.m scripts acting as sub-routines of the Wol_power.m script. A fourth file entitled data.xls must also be included in the same folder as the script files, formatted as per the template provided below.

**Wol_power.m coding information**

%Wol_power.m

%

% Power calculations for Serbus lab Wolbachia diet study.

% Randomization samples with replacement.

[data,txt] = xlsread('data.xls'); % read in Excel file.

alpha = 0.01;

nreps = 10000; % number of subsamples

nmin = 3; % smallest sample

nmax = size(data,1); % largest sample

stepsize = 1; % step size

GSC_control = data(:,1);

GSC_enriched = data(:,2);

stage4_control = data(:,3);

stage4_enriched = data(:,4);

stage10_control = data(:,5);

stage10_enriched = data(:,6);

figure(1)

Lauraplot((data), txt)

grid on

%xvar = GSC_control;

%yvar = GSC_enriched;

%xvar = stage4_control;

%yvar = stage4_enriched;

xvar = stage10_control;

yvar = stage10_enriched;

prob_rej_H0 = zeros(nmax,1);

n_array = zeros(nmax,1);

for n = nmin : stepsize : nmax % n is the size of the subsample

Parray = zeros(nmax,1);

Harray = zeros(nmax,1);

for k = 1:nreps

irand = rand(nmax,1);

%[a,i] = sort(irand); % find index i to sample without replacement

i = round(nmax * irand + 0.5); % find indeces i to sample with replacement

picks = i(1:n); % randomized indeces, the right number of them

x = xvar(picks);

irand = rand(n,1);

% [a,i] = sort(irand); % sample without replacement

i = round(nmax * irand + 0.5); % find indeces i to sample with replacement

picks = i(1:n); % randomized indeces, the right number of them

y = yvar(picks);

%[P,H] = ranksum(x,y); % rank sum test (Mann-Whitney)

[H,P] = ttest2(x,y,'vartype','unequal', 'alpha',alpha); % 2 independent samples t-test, unequal variance

Parray(k) = P;

Harray(k) = H;

end

prob_rej_H0(n) = sum(Harray)/nreps; % probability of rejecting H0 for this data set and sample size

n_array(n) = n;

disp(n)

end

figure(2)

plot(n_array, prob_rej_H0, 'o')

xlabel('sample size','FontSize',14)

ylabel('prob reject H0','FontSize',14)

title('Wol_power, alpha=0.01','Fontsize',14)

axis([0,15,0,1]);

grid on

hold off

**sem.m coding information**

function se = sem(x)

% function se = sem(x)

%

% calculates the standard error of the mean

% ignores NaN values

%

% PKS, 25 Mar 2007

%x = x(~isnan(x));

%se = std(x) / ((length(x)) ^0.5);

if (size(x,1)>1) & (size(x,2)>1)

disp('SEM requires a vector. Will not work with arrays')

return

end

se = nanstd(x) / (length(x(~isnan(x))) ^0.5);

**Lauraplot.m coding information**

function plotpops(data, varnames)

% function Lauraplot(data, varnames)

% PKS 30 Jan 2007

% Plots data from multiple populations and standard deviations.

% Data should be in colummns, one population each.

% Varnames (names of variables) is a cell array.

%% define population variables

ncols = size(data,2);% figure out how many columns we have in our data set

%data = data * 0.001;

% define false X variables

xa = ones(size(data));

for dog = 1:ncols

xa(:,dog) = dog;

end

% find population parameters

means = nanmean(data);

sems = zeros(size(means));

for i = 1:ncols

sems(:,i) = sem(data(:,i));

end

%% Graphics Section

% some user-defined graphics constants

plotstring = 'ok';

%plotstring = '+b';

errorbarstring = '-k';

%errorbarstring = '-b';

width = 0.10; % width of mean line

jitter_size = 0.05; % width of jitter for pseudo x variables

xoffset = 0.15; % separation between raw data and mean line

%add jitter to false X

jitter = jitter_size * 2 * rand(size(xa)) - jitter_size;

xajittered = xa + jitter;

% create error bar coordinates

ymeans = [means;means]; % create horizontal line coords for plotting means

xmeans = ones(size(ymeans));

ysems = [means - sems ; means + sems];

xsems = ones(size(ysems));

for i = 1:ncols

xmeans(1,i) = i - width/2;

xmeans(2,i) = i + width/2;

xsems(:,i) = i;

end

for i = 1:ncols

xmeans(1,i) = i - width/2;

xmeans(2,i) = i + width/2;

xsems(:,i) = i;

end

% plotting

semilogy(xajittered, data, plotstring)

hold on

%semilogy(xmeans+xoffset, ymeans, errorbarstring, 'linewidth',2); % means

%semilogy(xsems+xoffset, ysems, errorbarstring, 'linewidth', 2); % error bars (sems)

set(gca, 'Xtick', 1:ncols, 'XTickLabel', varnames); % put correct labels on x axis

hold off

maxdata = max(max(data));

mindata = min(min(data));

datarange = maxdata - mindata;

% extend y axis by 10% above & below so extreme data remain visible

maxy = maxdata + datarange * 0.1;

miny = mindata - datarange * 0.1;

ylim([miny, maxy])

minx = 0.7;

maxx = ncols + 0.4;

xlim([minx, maxx])

**data.xls template**

| GSC control | GSC enriched | stage4 control | stage4 enriched | stage10 control | stage10 enriched |
| --- | --- | --- | --- | --- | --- |
| 89 | 56 | 1085 | 1004 | 27270 | 7908 |
| 96 | 63 | 1107 | 1406 | 22650 | 12000 |
| 67 | 52 | 1714 | 1509 | 18382 | 5564 |
| 125 | 53 | 1123 | 1157 | 23420 | 9668 |
| 113 | 63 | 1226 | 1592 | 19522 | 9420 |
| 96 | 67 | 944 | 1440 | 17608 | 9836 |
| 116 | 46 | 1536 | 1261 | 27856 | 8628 |
| 79 | 54 | 1175 | 1262 | 21776 | 8236 |
| 42 | 63 | 1063 | 1399 | 26190 | 8802 |
| 47 | 46 | 1438 | 1233 | 30748 | 8968 |
| 72 | 62 | 1864 | 1192 | 33318 | 6910 |
| 48 | 63 | 1212 | 1370 | 20538 | 6474 |
| 87 | 45 | 1090 | 1306 | 26144 | 5930 |
| 28 | 55 | 1130 | 1088 | 22894 | 5274 |
| 52 | 55 | 1490 | 1066 | 15332 | 8244 |
| 79 | 55 | 1175 | 1262 | 22894 | 8244 |
